# Leptin in Sarcopenic Visceral Obesity: Possible Link between Adipocytes and Myocytes

**DOI:** 10.1371/journal.pone.0024633

**Published:** 2011-09-09

**Authors:** Katsuhiko Kohara, Masayuki Ochi, Yasuharu Tabara, Tokihisa Nagai, Michiya Igase, Tetsuro Miki

**Affiliations:** 1 Department of Geriatric Medicine, Ehime University Graduate School of Medicine, Ehime, Japan; 2 Department of Basic Medical Research and Education, Ehime University Graduate School of Medicine, Ehime, Japan; 3 Proteo-Medicine Research Center, Ehime University Graduate School of Medicine, Ehime, Japan; University of Padova, Medical School, Italy

## Abstract

The combination of sarcopenia, age-related loss of muscle strength and mass, and obesity has been recognized as a new category of obesity among the elderly. Given that leptin has been hypothesized to be involved in the pathogenesis of sarcopenic obesity, we investigated the relationship between plasma leptin levels and thigh muscle sarcopenia and visceral obesity. Thigh muscle cross-sectional area (CSA) and visceral fat area were measured using computed tomography as indices for muscle mass and visceral fat, respectively, in 782 middle-aged to elderly subjects (303 men and 479 women), participating in a medical check-up program. Visceral obesity was defined as visceral fat area >100 cm^2^, and sarcopenia was defined as < (one standard deviation − mean of thigh muscle CSA/body weight of young subjects [aged <50 years]).

Thigh muscle CSA was significantly and negatively associated with plasma levels of leptin in both men (β = -0.28, p<0.0001) and women (β = -0.20, p<0.0001), even after correcting for other confounding parameters, including age, body weight, body height, visceral fat area, blood pressure, homeostatic model assessment index, and high sensitive C reactive protein. Subjects were divided into four groups based on presence or absence of sarcopenia or visceral obesity. Plasma levels of leptin were higher in subjects with sarcopenic visceral obesity than in those with either sarcopenia or visceral obesity alone. These findings indicate that sarcopenic visceral obesity is a more advanced, and suggest that leptin may link visceral obesity and sarcopenia.

## Introduction

Obesity, particularly visceral obesity, is a global pandemic known to be related to numerous pathological conditions, including metabolic syndrome, diabetes, hypertension, and cardiovascular disease [1]–[3]. Sarcopenia, which is the age-related loss of muscle mass, is another concern in today's aging society [4]. In particular, sarcopenia in the legs leads to functional impairments such as postural instability, falling, and frailty, eventually culminating in loss of independence and death [**5**]. Recent studies have turned attention to sarcopenic-obesity, a deadly combination of the above two conditions which synergistically deteriorates metabolic disorders as well as functional abnormalities [1]–[3]. Prevalence of sarcopenic-obesity is increasing in industrialized countries due to increased prevalence of obesity itself and sarcopenia in obese subjects [1]–[3], [6]. Determining the underlying mechanisms linking sarcopenia and obesity is therefore crucial to enacting effective intervention protocols.

Adipocyte-myocyte crosstalk via adipokines is believed to underlie the pathophysiology of obesity-related disorders [7]. Leptin is released from adipocytes and exerts a range of pathological and physiological actions on a number of organs, including skeletal muscle [8]. Specifically, leptin acts on skeletal muscles to stimulate lipolysis and insulin sensitivity [9]. Serum levels of leptin increase with fat accumulation [8]–[10]. Although its central effect decreases in this state, leptin maintains its peripheral effect, subsequently leading to stimulation of the sympathetic nervous system and platelet aggregation, effects which may underlie the connection between obesity and cardiovascular diseases such as hypertension [11] and atherosclerosis [10].

Leptin receptors have been shown to be down-regulated by leptin itself or via insulin resistance [10]. Since skeletal muscle is the major site of glucose consumption, presence of sarcopenia is another risk factor in developing insulin resistance [1]–[3]. Further, leptin receptor numbers may be reduced along with muscle mass in patients with sarcopenia, possibly resulting in further increases in plasma leptin concentration. These findings suggest that leptin may play a greater role in sarcopenic obesity than in simple obesity. However, studies evaluating the relationship between sarcopenia and circulating leptin levels have thus far drawn inconclusive results due to a small patient population [12]–[14].

Here, we conducted a cross-sectional study to investigate relationships between plasma leptin levels and sarcopenia and obesity in 782 middle-aged to elderly subjects participating in a medical check-up program in Japan.

## Methods

### Study subjects

Subjects were independent middle-aged to elderly persons recruited from among consecutive visitors to the Anti-Aging Center at Ehime University Hospital from March 2006 to March 2009. They attended the voluntary medical check-up program, “Anti-Aging Doc,” a program provided to general residents of Ehime Prefecture, Japan, specifically designed to evaluate aging-related disorders, including atherosclerosis, cardiovascular disease, physical function, and mild cognitive impairment [15], [16]. Of the 844 consecutive patients initially approached, a total of 782 who agreed with the study aims and protocols, gave written consent to all procedures, and were free of any history of symptomatic cardiovascular events including peripheral arterial diseases, stroke, coronary heart disease, and congestive heart failure were analyzed. All participants were physically independent in their daily lives. The series of studies to which the present study belongs was approved by the Ethics Committee of Ehime University Graduate School of Medicine.

### Measurement of femoral muscle cross-sectional area and visceral fat area

**Figure 1:**
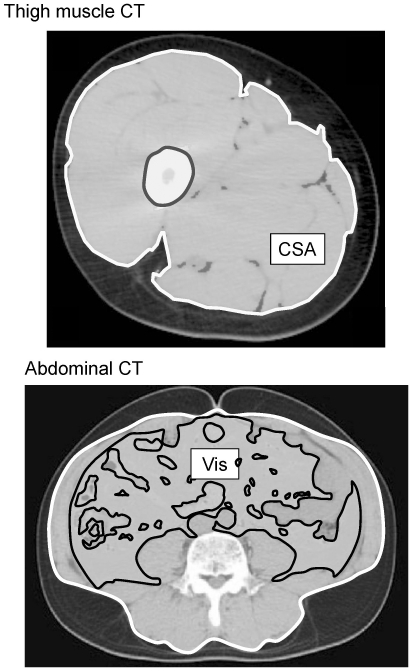
Thigh muscle CT (top) at the mid-thigh level and abdominal CT (bottom) at umbilicus level. Thigh muscle cross-sectional area (CAS) and visceral fat area (Vis) were obtained.

Femoral muscle cross-sectional area (CSA) was measured using computed tomography (CT; LightSpeed VCT; GE Healthcare, Tokyo, Japan) at the mid-thigh, measured as the midpoint from the inguinal crease to the proximal pole of the patella [16]. The muscle CSA (cm^2^) was computed using an attenuation range of 0 to 100 Hounsfield units. Visceral fat area was measured using CT at the level of the umbilicus, with attenuation ranging from 150 to 50 Hounsfield units. CT images were obtained with a minimal slice width of 5 mm and analyzed using OsiriX software (OsiriX Foundation, Geneva, Switzerland) (**Fig. 1**) [17]. All images were analyzed by a single investigator under blinded conditions (O.M.).

### Definition of visceral obesity and thigh muscle sarcopenia

**Figure 2:**
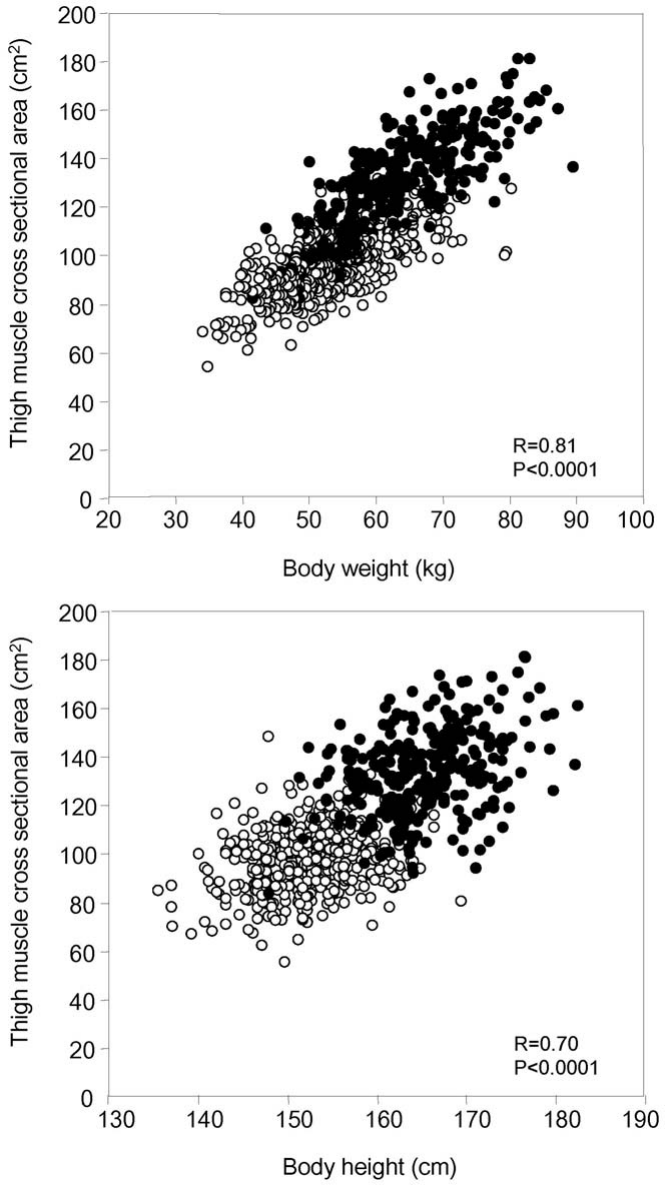
Scatter plots between thigh muscle cross-sectional area and body weight (top) and body height (bottom). •: men, ○: women.

Visceral obesity was defined as a visceral fat area >100 cm^2^ in both men and women, in accordance with the Japanese criterion defining metabolic syndrome [18]. The category of “overweight,” defined as having a BMI>25 kg/m^2^, was also evaluated as an obesity index. Since thigh muscle CSA showed a strong association with body weight (BW) (**Fig. 2**), thigh muscle CSA was corrected by body weight (CSA/BW) as a sarcopenic index of BW burden thigh muscle mass. Sarcopenia was defined in both men and women as having CSA/BW within one standard deviation value of the CSA/BW distribution in subjects aged <50 years among study participants [8], [19].

### Biochemical determination

To determine biochemical variables, blood samples were obtained from each subject after fasting overnight for at least 11 h. jePlasma concentrations of leptin were determined using a commercially available RIA kit (Leptin HL-81K; Linco Research, Inc., St. Charles, MO, USA) [20]. Coefficients of variation in our inter- and intra-assay were 7.2% and 4.1%, respectively.

### Statistical analysis

Values are expressed as the mean ± standard deviation unless otherwise specified. Subjects were divided into three groups according to their thigh muscle CSA and abdominal visceral fat area. Since body composition profiles differ between men and women, tertiles were obtained separately for both sexes. Factors independently associated with plasma levels of leptin were assessed using multiple regression analysis with two models, model 1 with anthropometric parameters and model 2 with further parameters possibly related to leptin levels. Plasma levels of leptin in all tertiles of visceral fat area and thigh muscle CSA were also evaluated via multiple regression analysis with age, BH, and BW.

Finally, subjects were divided into four groups in the presence and absence of visceral obesity and thigh muscle sarcopenia. The four groups—established base on being overweight or not and presence or absence of thigh muscle sarcopenia—were also evaluated.

Differences in numeric variables among groups were assessed using analysis of variance (ANOVA) followed by Bonferroni correction for multiple comparison test, and differences in frequency were assessed using the chi-squared test. All analyses were conducted using commercially available statistical software (JMP ver. 8.0; SAS Institute, Cary, NC, USA), with p<0.05 considered statistically significant.

## Results

### Plasma leptin with visceral fat area and thigh muscle CSA

Clinical characteristics of the study population are shown in **Table 1**. Both thigh muscle CSA and abdominal visceral fat area were found to be significantly related to BW (r = 0.80, p<.0001, and r = 0.70, p<.0001, respectively) (**Fig. 2**). Thigh muscle CSA was also significantly related to body height (BH) (r = 0.70, p<0.0001)

**Table 1:** Clinical characteristics of study population.

	Men	Women	p
N	303	479	
Age, years	67.9±8.5	66.3±8.2	0.0045
Body height, cm	164.9±5.9	152.2±5.3	< .0001
Body weight, kg	64.5±8.8	52.7±7.8	< .0001
Body mass index, kg/m^2^	23.7±2.9	22.8±3.2	< .0001
Waist, cm	86.0±8.0	82.5±9.2	< .0001
Hip, cm	92.5±5.4	90.1±5.7	< .0001
Waist/hip ratio	0.93±0.05	0.91±0.07	0.0007
Visceral fat area, cm^2^	128.4±64.8	87.4±51.6	< .0001
Thigh muscle CSA, cm^2^	133.5±17.7	96.1±13.3	< .0001
Thigh muscle CSA/BW, cm^2^/kg	2.1±0.2	1.8±0.2	< .0001
Systolic blood pressure, mmHg	138.1±19.3	136.1±19.7	0.16
Diastolic blood pressure, mmHg	79.7±11.4	76.9±11.1	0.0013
Heart rate, beats/min	64.7±11.3	67.2±10.1	0.001
Total cholesterol, mg/dl	207.7±32.5	225.6±35.7	< .0001
HDL cholesterol, mg/dl	61.5±17.7	73.0±18.4	< 0.001
Triglyceride, mg/dl	111.8±57.7	101.3±55.0	0.0064
Fasting glucose, mg/dl	107.4±18.5	100.5±15.0	< .0001
Insulin, μU/ml	5.98±3.72	5.49±3.94	0.069
HbA1c, %	5.6±0.7	5.5±0.6	0.062
HOMA-R	1.6±1.2	1.4±1.2	0.0077
hs-CRP, mg/dl	0.11±0.12	0.09±0.12	0.12
WBCs, /μL	5698±1564	5177±1288	< .0001
Medication, n (%)			
Hypertension	99 (33)	127 (27)	0.087
Dyslipidemia	46 (15)	117 (24)	0.003
Diabetes	21 (7)	22 (5)	0.17
Smoking, current/past/never smoker, n	39/187/77	11/25/443	< .0001

CSA, cross sectional area; BW, body weight; HDL, high density lipoprotein; HbA1c, hemoglobin A1c; HOMA, homeostatic model assessment; hs-CRP, high-sensitivity C-reactive protein; WBCs, white blood cells, P indicates p values between men and women.

**Figure 3:**
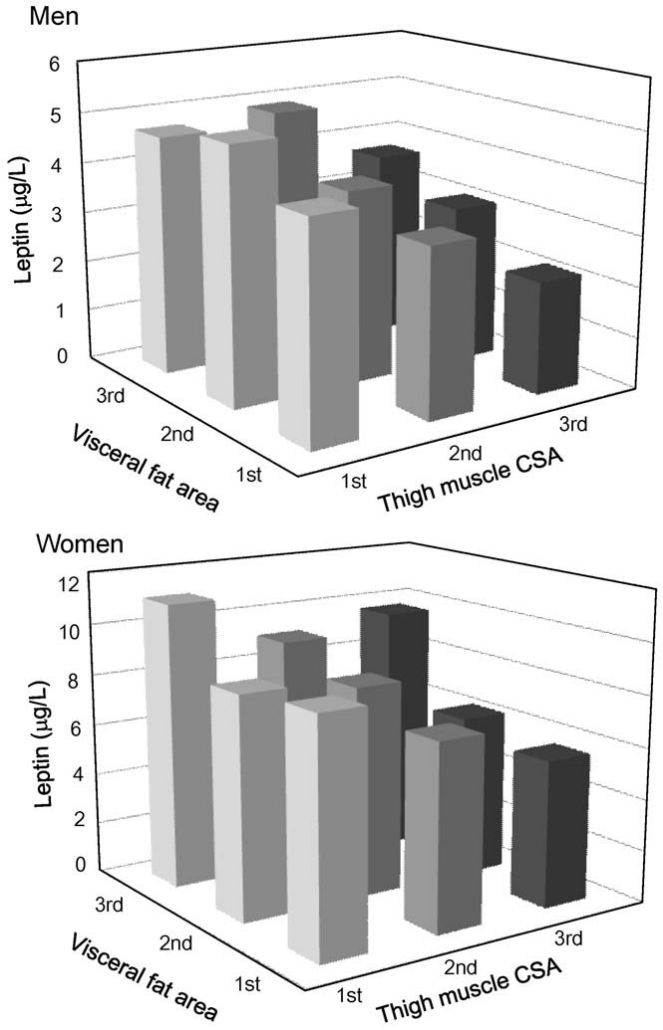
Plasma leptin levels in tertiles of visceral fat area and thigh muscle cross-sectional area (CSA). Multiple regression analysis was performed for plasma leptin concentration with the following parameters in each sex: age, body weight, body height, tertiles of visceral fat area, tertiles of thigh muscle CSA, and interaction and interaction between visceral fat and thigh muscle tertiles. All values are mean values. **Men**: Visceral fat area tertile: F = 5.1, p = 0.007. Thigh muscle CSA tertile: F = 8.5, p = 0.0003. Interaction: F = 1.5, p = 0.33. **Women**: Visceral fat area tertile: F = 7.6, p = 0.0006. Thigh muscle CSA tertile: F = 5.2, p = 0.006. Interaction: F = 1.1, p = 0.34.

Plasma levels of leptin in the tertiles of visceral fat area and thigh muscle CSA after correction for age, BH and BW are shown in **Figure 3**. In both men and women, plasma levels of leptin were significantly and positively related to visceral fat area and inversely related to thigh muscle CSA.

### Multiple regression analysis for plasma levels of leptin

We conducted multiple regression analyses to investigate whether or not thigh muscle CSA is independently associated with plasma levels of leptin (**Table 2**). After correction for possible confounding parameters, including age, BH, BW, abdominal visceral fat area, systolic and diastolic blood pressure (BP), levels of total cholesterol, high-density lipoprotein cholesterol, triglyceride, HbA1c, Homeostatic Model Assessment as an index of insulin resistance (HOMA-R), hs-CRP, and white blood cell count, thigh muscle CSA was found to be independently, significantly, and negatively related to plasma leptin levels in both men and women.

**Table 2:** Multiple regression analysis for plasma leptin concentration.

	Model 1				Model 2			
	Men		Women		Men		Women	
	β	p	β	p	β	P	β	P
Age, years	-0.06	0.25	0.01	0.79	0	0.98	-0.01	0.82
Body height, cm	-0.25	<.0001	-0.17	0.0002	-0.15	0.005	-0.13	0.002
Body weight, kg	0.71	<.0001	0.54	<.0001	0.55	<.0001	0.47	<.0001
Visceral fat area, cm^2^	0.28	<.0001	0.28	<.0001	0.23	0.0004	0.23	<.0001
Thigh muscle CSA	-0.33	<.0001	-0.2	<.0001	-0.27	<.0001	-0.2	<.0001
Systolic BP, mmHg					-0.1	0.07	0.01	0.88
Diastolic BP, mmHg					0.05	0.34	0	0.94
Total cholesterol, mg/dl					0.07	0.09	0.02	0.7
HDL cholesterol, mg/dl					0.08	0.1	0.01	0.77
Triglyceride, mg/dl					-0.07	0.14	-0.01	0.75
HbA1c, %					-0.1	0.042	-0.04	0.37
HOMA-R					0.39	<.0001	0.25	<.0001
hs-CRP					0.01	0.71	0.02	0.54
WBCs					0.02	0.58	0.04	0.23
Antihypertensive drugs, yes = 1					-0.01	0.9	-0.01	0.74
Antidislipidemic drug use, yes = 1					0.09	0.037	0.03	0.46
Diabetic medication, yes = 1					-0.07	0.14	-0.07	0.12

CSA, cross sectional area; BW, body weight; HDL, high density lipoprotein; HbA1c, hemoglobin A1c; HOMA, homeostatic model assessment; hs-CRP, high-sensitivity C-reactive protein; WBCs, white blood cells.

### Plasma leptin in sarcopenic visceral obesity

**Figure 4:**
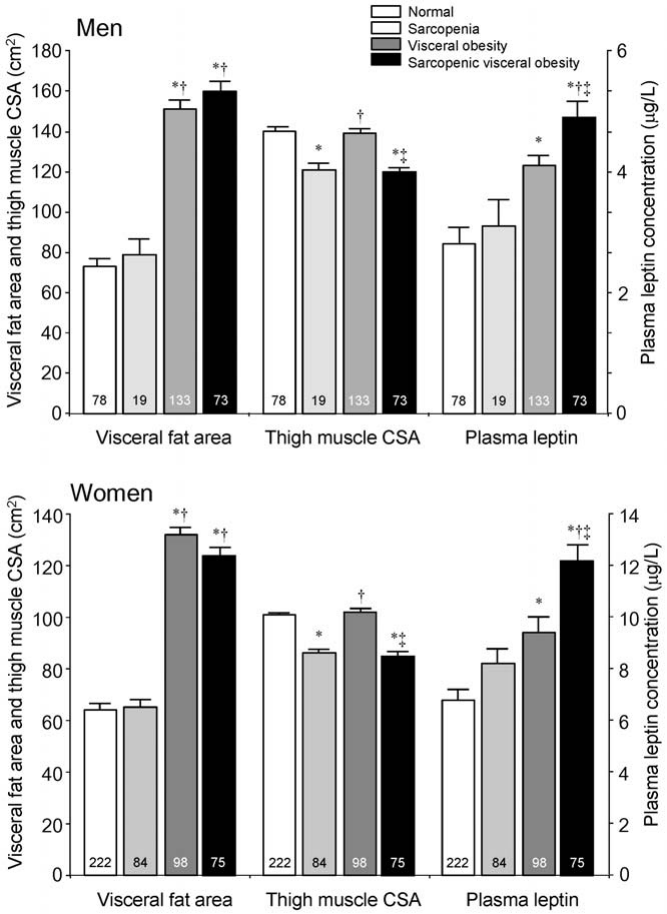
Visceral fat area, thigh muscle cross-sectional area (CSA) and plasma levels of leptin in subjects with sarcopenia, visceral obesity, and sarcopenic visceral obesity. Corrected for age and body weight. Values are mean ± standard error. *p<.05 vs. normal, †p<.05 vs. sarcopenic, ‡p<.05 vs. visceral obesity.

To explore the combined effect of sarcopenia and visceral obesity on plasma levels of leptin, four groups based on presence or absence of either or both thigh muscle sarcopenia and visceral obesity were compared. After adjustment for age and BW, levels of plasma leptin were found to be highest in both men and women among subjects with sarcopenic visceral obesity followed by simple obesity (**Table 3** **,** **4** **, and** **Fig. 4**).

**Table 3:** Clinical characteristics of subjects with thigh muscle sarcopenia, visceral obesity and sarcopenic visceral obesity in men.

	Normal	Sarcopenia	Visceral obesity	Sarcopenic visceral obesity	P
N	78	19	133	73	
Age, years	67.9±8.1	73.0±5.0*	65.7±8.2	70.5±9.3*^‡^	<.0001
Body Height^a^, cm	165.1±5.4	166.3±5.4	163.4±5.4	166.9±5.4*	0.0002
Body Weight^a^, kg	57.6±7.0	59.7±7.1	65.7±7.2^*†^	71.0±7.1^*†‡^	<.0001
Body mass index^a^, kg/m^2^	21.1±2.3	21.6±2.3	24.6±2.3^*†^	25.5±2.3^*†‡^	<.0001
Waist/hip ratio^a^	0.88±0.04	0.91±0.04	0.94±0.05^*†^	0.96±0.04^*†^	<.0001
Visceral fat area^a^, cm^2^	52.8±39.8	65.0±40.3	154.2±40.5^*†^	178.6±40.3^*†‡^	<.0001
Thigh muscle CSA^a^, cm^2^	127.9±14.0	113.2±14.2*	141.0±14.2*^†^	131.1±14.2^†‡^	<.0001
Thigh muscle CSA/BW^a^, cm^2^/kg	2.22±0.13	1.89±0.13*	2.15±0.13*^†^	1.85±0.13*^‡^	<.0001
Leptin^a^, μg/L	1.9±2.3	2.6±2.3*	4.3±2.3*^†^	5.7±2.3^*†‡^	<.0001
Systolic blood pressure^b^, mmHg	129.8±20.3	132.1±18.7	142.9±18.5*	139.7±20.5*	0.0001
Diastolic blood pressure^b^, mmHg	75.1±12.4	76.2±11.3	82.4±11.5*	80.4±12.0*	0.0004
Total cholesterol^b^, mg/dl (mmol/L)	203.6±36.1 (5.27±0.97)	195.1±32.3 (5.05±0.87)	211.9±33.0 (5.49±0.92)	207.4±35.6 (5.37±0.94)	0.15
HDL cholesterol^b^,mg/ml (mmol/L)	65.3±18.4	60.9±16.5	59.2±16.9^*^	61.6±18.2	0.12
	(1.69±0.44)	(1.58±0.44)	(1.53±0.46^*^)	(1.60±0.51)	
Triglyceride^b^, mg/dl (mmol/L)	86.9±60.0	95.2±53.8	125.5±54.8^*^	117.8±59.4^*^	0.0001
	(0.98±0.71)	(1.08±0.61)	(1.42±0.58^*^)	(1.33±0.77^*^)	
Fasting lucose^b^, mg/dl (mmol/L)	102.4±20.3	113.9±18.2	106.5±18.6	112.7±19.9^*^	0.0096
	(5.68±1.15)	(6.32±1.05)	(5.91±1.04)	(6.25±1.11^*^)	
Insulin^b^, μU/mL(pmol/L)	5.07±3.60	50.9±3.27	61.6±3.33	6.85±3.56^*^	0.036
	(35.4±24.7)	(35.4±24.4)	(43.1±24.2)	(47.9±23.9^*^)	
HbA1c^b^, %	5.4±0.7	5.7±0.7	5.6±0.7*	5.9±0.8^*†^	0.0009
HOMA-R^b^	1.30±1.24	1.39±1.13	1.65±1.15	1.99±1.20^*^	0.014
hs-CRP^b^, mg/dl	0.14±0.34	0.10±0.31	0.15±0.31	0.15±0.34	0.92
WBC^b^,/μl	5255±1713	6017±1574	5648±1568	6115±1700^*^	0.024

CSA, cross sectional area; BW, body weight; HDL, high density lipoprotein; HbA1c, hemoglobin A1c, HOMA, homeostatic model assessment;hs-CRP, high-sensitivity C-reactive protein; WBC, white blood cell.

a : corrected for age,

b : corrected for age and body weight.

**Table 4:** Clinical characteristics of subjects with thigh muscle sarcopenia, visceral obesity and sarcopenic visceral obesity in women.

	Normal	Sarcopenia	Visceral obesity	Sarcopenic visceral obesity	P
N	78	19	133	73	
Age, years	67.9±8.1	73.0±5.0*	65.7±8.2	70.5±9.3*^‡^	<.0001
Body Height^a^, cm	165.1±5.4	166.3±5.4	163.4±5.4	166.9±5.4*	0.0002
Body Weight^a^, kg	57.6±7.0	59.7±7.1	65.7±7.2^*†^	71.0±7.1^*†‡^	<.0001
Body mass index^a^, kg/m^2^	21.1±2.3	21.6±2.3	24.6±2.3^*†^	25.5±2.3^*†‡^	<.0001
Waist/hip ratio^a^	0.88±0.04	0.91±0.04	0.94±0.05^*†^	0.96±0.04^*†^	<.0001
Visceral fat area^a^, cm^2^	52.8±39.8	65.0±40.3	154.2±40.5^*†^	178.6±40.3^*†‡^	<.0001
Thigh muscle CSA^a^, cm^2^	127.9±14.0	113.2±14.2*	141.0±14.2*^†^	131.1±14.2^†‡^	<.0001
Thigh muscle CSA/BW^a^, cm^2^/kg	2.22±0.13	1.89±0.13*	2.15±0.13*^†^	1.85±0.13*^‡^	<.0001
Leptin^a^, μg/L	1.9±2.3	2.6±2.3*	4.3±2.3*^†^	5.7±2.3^*†‡^	<.0001
Systolic blood pressure^b^, mmHg	129.8±20.3	132.1±18.7	142.9±18.5*	139.7±20.5*	0.0001
Diastolic blood pressure^b^, mmHg	75.1±12.4	76.2±11.3	82.4±11.5*	80.4±12.0*	0.0004
Total cholesterol^b^, mg/dl (mmol/L)	203.6±36.1 (5.27±0.97)	195.1±32.3 (5.05±0.87)	211.9±33.0 (5.49±0.92)	207.4±35.6 (5.37±0.94)	0.15
HDL cholesterol^b^,mg/ml (mmol/L)	65.3±18.4	60.9±16.5	59.2±16.9^*^	61.6±18.2	0.12
	(1.69±0.44)	(1.58±0.44)	(1.53±0.46^*^)	(1.60±0.51)	
Triglyceride^b^, mg/dl (mmol/L)	86.9±60.0	95.2±53.8	125.5±54.8^*^	117.8±59.4^*^	0.0001
	(0.98±0.71)	(1.08±0.61)	(1.42±0.58^*^)	(1.33±0.77^*^)	
Fasting lucose^b^, mg/dl (mmol/L)	102.4±20.3	113.9±18.2	106.5±18.6	112.7±19.9^*^	0.0096
	(5.68±1.15)	(6.32±1.05)	(5.91±1.04)	(6.25±1.11^*^)	
Insulin^b^, μU/mL (pmol/L)	5.07±3.60	50.9±3.27	61.6±3.33	6.85±3.56^*^	0.036
	(35.4±24.7)	(35.4±24.4)	(43.1±24.2)	(47.9±23.9^*^)	
HbA1c^b^, %	5.4±0.7	5.7±0.7	5.6±0.7*	5.9±0.8^*†^	0.0009
HOMA-R^b^	1.30±1.24	1.39±1.13	1.65±1.15	1.99±1.20^*^	0.014
hs-CRP^b^, mg/dl	0.14±0.34	0.10±0.31	0.15±0.31	0.15±0.34	0.92
WBC^b^,/μl	5255±1713	6017±1574	5648±1568	6115±1700^*^	0.024

CSA, cross sectional area; BW, body weight; HDL, high density lipoprotein; HbA1c, hemoglobin A1c, HOMA, homeostatic model assessment; hs-CRP, high-sensitivity C-reactive protein; WBC, white blood cell.

a : corrected for age,

b : corrected for age and body weight.

Tables 3 and 4 also summarize metabolic and inflammatory parameters in the four groups. We noted no differences in blood pressure or total cholesterol among in men or women in any groups. However, insulin resistance parameters—including fasting insulin and HOMA-R—and numbers of WBCs were significantly increased among patients with sarcopenic visceral obesity compared with normal men and women.

### Plasma leptin in sarcopenic overweight individuals

**Figure 5:**
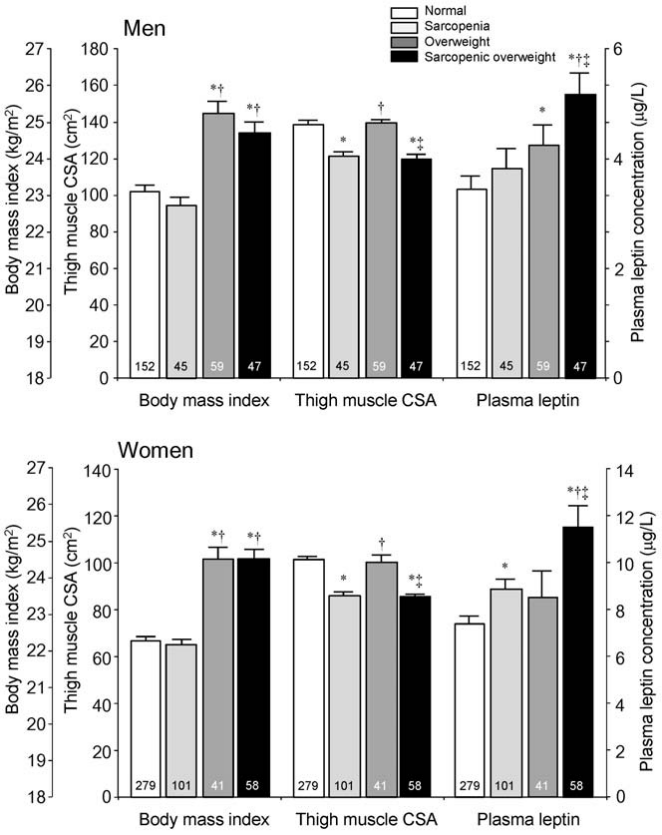
Body mass index, thigh muscle cross-sectional area (CSA) and plasma leptin levels in sarcopenic, overweight, and sarcopenic overweight subjects. Corrected for age and body weight. Values are mean ± standard error. *p<.05 vs. normal, †p<.05 vs. sarcopenic, ‡p<.05 vs. overweight subjects.

Plasma levels of leptin in normal, sarcopenic, overweight, and sarcopenic overweight groups are depicted in Fig 5. Similar to findings in individuals with sarcopenic visceral obesity, plasma leptin levels after adjustment for age and BW were highest in both male and female sarcopenic overweight subjects compared with all other three groups.

## Discussion

Here, we found that plasma leptin levels were significantly, independently, and negatively related to thigh muscle CSA in middle-aged to elderly men and women. Further, in both men and women, leptin levels were found to be higher in subjects with both thigh muscle sarcopenia and visceral obesity or being overweight than in those with only one abnormality. These findings suggest that plasma leptin level may be associated with not only visceral fat but also skeletal muscle mass.

Previous studies investigating the association between leptin and sarcopenia have been inconclusive. Gómez et al. [12] noted a positive relationship between serum levels of leptin and fat-free mass in both men and women. However, after correction for other confounding parameters, the authors ultimately concluded that free-fat mass did not contribute to leptin levels [12]. Hubbard et al. [13] reported low leptin levels in frail elderly subjects with reduced mid-arm muscle area, and they speculated it was due to low body fat [13]. In contrast, in their study involving 45 healthy elderly subjects, Waters et al. [14] reported a negative correlation between appendicular skeletal muscle mass as measured using a dual energy X-ray absorptiometer and leptin levels after correction with body fat. We confirmed these authors' findings in a much larger population using the more specifically quantified thigh muscle CSA.

We further observed that subjects with sarcopenic visceral obesity had higher leptin levels than in any other groups, including visceral obese subjects without sarcopenia, findings which ran counter to observations by Waters et al. [14]. After correction for fat mass, these authors noted higher concentrations of leptin among subjects with sarcopenia than those with sarcopenic obesity [14]. The differing results between our study and that of Waters et al. may be due to differing definitions of sarcopenia and obesity. Waters et al. used appendicular muscle mass corrected by BH^2^ (m^2^) as an index for sarcopenia and defined sarcopenia as two SD below the sex-specific mean values for young adults aged 18-40 years. In the present study, we used thigh muscle CSA corrected by BW, since thigh muscle CSA showed higher associations with BW than BH^2^ both in men (r = 0.73 vs. r = 0.35, p<0.0001) and women (r = 0.66 vs. r = 0.27, p<0.0001). Further we defined visceral obesity as visceral fat area >100 cm^2^, in accordance with the Japanese guidelines for metabolic syndrome [19].

Since visceral fat area was higher in sarcopenic obesity group than single obesity group after correction for age, the difference in plasma leptin between the two groups could be influenced by visceral fat area itself rather than thigh muscle CSA. Accordingly we performed further adjustment with BW. After adjustment, there was no significant difference in visceral fat area between the two groups, while plasma leptin was significantly higher in sarcopenic obesity than single obesity group. These findings together with the result of multiple regression analyses indicate that thigh muscle CSA negatively related to plasma leptin levels independently of visceral fat area. Further, in a separate analysis we evaluated the effect of BMI-defined overweight condition on plasma levels of leptin. After correction for age and BW, both male and female sarcopenic overweight individuals (BMI>25 kg/m^2^) had the highest plasma leptin levels of all groups measured. These findings indicate that the effect of sarcopenia on plasma levels of leptin is not only independent of visceral fat area but also BMI.

Determining the mechanisms behind the negative association between thigh muscle CSA and plasma leptin is beyond the scope of the present study. However, leptin is known to stimulate inflammation [7]–[10], and serum leptin levels have also been shown to be negatively correlated with IGF-1 levels and testosterone [21]. Given that these factors are known to play roles in development of sarcopenia [1]–[3], leptin may also play a causative role in development of sarcopenia. Exogenous leptin has also been shown to reduce protein synthesis in myocytes [22]. In contrast, a recent study demonstrated that leptin treatment significantly increased hindlimb muscle mass and extensor digitorum longus fiber size in aged mice [23]. Given that obesity is associated with reduced number and function of leptin receptors in leg muscle [24], these present and previous findings suggest that impaired action of leptin in skeletal muscle may also induce development of sarcopenia.

Several limitations to our study warrant mention. The cross-sectional nature of our research hampered to determine that obesity and sarcopenia affect plasma leptin levels. In addition, our study subjects were free from any symptomatic cardiovascular diseases, and few had BMI>30; as such, our findings may not be easily extrapolated to different ethnicities, younger generations, or subjects with more severe obesity. A longitudinal study involving a more divergent population will be necessary to address these points.

In summary, thigh muscle CSA was negatively associated with plasma leptin concentration in middle-aged to elderly subjects, independent of known confounding factors, including age, BW, HOMA index, and visceral fat area. These findings further highlight the importance of sarcopenia in understanding the pathophysiological aspect of obesity in the elderly population.
